# Supplementary material for: Automated structure refinement of macromolecular assemblies from cryo-EM maps using Rosetta
Source: eLife. 2016 Sep 26;5:e17219. doi: 10.7554/eLife.17219 (PMC5115868; doi:10.7554/eLife.17219)
Supplement: Figure 6—source data 1. — DOI: http://dx.doi.org/10.7554/eLife.17219.014 [file elife-17219-fig6-data1.docx]

Figure 6-source data 1. Mitoribosome per-chain refinement results

| Chain ID | | iFSC | | |  | MolProbity  Score | | | | Ramachandran Favored | | | EMRinger Score | | | |
| --- | --- | --- | --- | --- | --- | --- | --- | --- | --- | --- | --- | --- | --- | --- | --- | --- |
| 0 |  | 0.630 | / | 0.623 |  | 2.85 | / | 0.84 |  | 86.17 | / | 98.94 | 1.74 | / | 2.60 |  |
| 1 |  | 0.608 | / | 0.607 |  | 2.48 | / | 0.77 |  | 92.00 | / | 96.00 | 0.86 | / | 2.75 |  |
| 2 |  | 0.698 | / | 0.667 |  | 1.81 | / | 0.84 |  | 97.56 | / | 95.12 | 1.68 | / | 4.15 |  |
| 3 |  | 0.719 | / | 0.676 |  | 2.11 | / | 1.06 |  | 94.62 | / | 94.62 | 3.11 | / | 5.09 |  |
| 4 |  | 0.682 | / | 0.643 |  | 2.29 | / | 0.50 |  | 100.00 | / | 100.00 | 4.32 | / | 4.40 |  |
| 5 |  | 0.653 | / | 0.639 |  | 2.76 | / | 1.31 |  | 89.40 | / | 94.02 | 2.80 | / | 2.68 |  |
| 6 |  | 0.530 | / | 0.496 |  | 2.49 | / | 1.39 |  | 87.54 | / | 90.73 | 2.54 | / | 2.20 |  |
| 7 |  | 0.583 | / | 0.567 |  | 2.27 | / | 0.98 |  | 91.47 | / | 94.19 | 2.22 | / | 2.14 |  |
| 8 |  | 0.344 | / | 0.334 |  | 1.67 | / | 0.80 |  | 96.36 | / | 98.18 | 2.03 | / | 0.68 |  |
| 9 |  | 0.581 | / | 0.537 |  | 1.91 | / | 1.61 |  | 88.57 | / | 92.38 | 2.25 | / | 2.19 |  |
| D |  | 0.679 | / | 0.646 |  | 2.17 | / | 1.39 |  | 94.87 | / | 93.16 | 3.50 | / | 4.11 |  |
| E |  | 0.717 | / | 0.688 |  | 2.58 | / | 1.35 |  | 90.54 | / | 94.63 | 2.40 | / | 3.29 |  |
| F |  | 0.691 | / | 0.666 |  | 2.50 | / | 1.02 |  | 91.94 | / | 93.55 | 3.71 | / | 3.44 |  |
| H |  | 0.580 | / | 0.581 |  | 2.67 | / | 1.04 |  | 87.10 | / | 96.77 | 2.55 | / | 2.79 |  |
| I |  | 0.408 | / | 0.457 |  | 2.68 | / | 0.94 |  | 92.86 | / | 97.40 | 2.02 | / | 1.26 |  |
| J |  | 0.258 | / | 0.328 |  | 2.73 | / | 1.40 |  | 83.33 | / | 92.75 | -0.12 | / | 1.41 |  |
| K |  | 0.684 | / | 0.655 |  | 2.35 | / | 0.98 |  | 89.71 | / | 94.86 | 2.04 | / | 3.61 |  |
| L |  | 0.675 | / | 0.651 |  | 2.42 | / | 1.18 |  | 88.50 | / | 94.69 | 0.26 | / | 1.63 |  |
| M |  | 0.686 | / | 0.654 |  | 2.55 | / | 1.47 |  | 89.82 | / | 93.33 | 2.04 | / | 2.43 |  |
| N |  | 0.662 | / | 0.649 |  | 2.25 | / | 1.16 |  | 91.63 | / | 92.61 | 2.53 | / | 2.89 |  |
| O |  | 0.676 | / | 0.660 |  | 2.91 | / | 1.23 |  | 88.67 | / | 96.67 | 1.55 | / | 3.10 |  |
| P |  | 0.642 | / | 0.621 |  | 2.72 | / | 0.99 |  | 89.15 | / | 95.35 | 2.22 | / | 4.80 |  |
| Q |  | 0.671 | / | 0.659 |  | 2.76 | / | 0.97 |  | 89.50 | / | 96.00 | 2.90 | / | 3.86 |  |
| R |  | 0.690 | / | 0.664 |  | 2.31 | / | 0.95 |  | 92.75 | / | 95.65 | 4.19 | / | 4.51 |  |
| S |  | 0.656 | / | 0.627 |  | 2.31 | / | 1.01 |  | 93.51 | / | 96.10 | 3.84 | / | 2.02 |  |
| T |  | 0.682 | / | 0.660 |  | 2.49 | / | 0.91 |  | 92.68 | / | 96.95 | 2.79 | / | 2.50 |  |
| U |  | 0.662 | / | 0.653 |  | 2.59 | / | 1.06 |  | 88.07 | / | 96.33 | 3.20 | / | 4.32 |  |
| V |  | 0.519 | / | 0.490 |  | 2.17 | / | 1.28 |  | 87.98 | / | 94.54 | 1.54 | / | 1.93 |  |
| W |  | 0.687 | / | 0.671 |  | 2.61 | / | 0.95 |  | 97.14 | / | 96.19 | 3.82 | / | 6.28 |  |
| X |  | 0.632 | / | 0.603 |  | 2.56 | / | 0.50 |  | 91.29 | / | 98.34 | 2.12 | / | 1.74 |  |
| Y |  | 0.633 | / | 0.607 |  | 2.46 | / | 1.07 |  | 90.23 | / | 95.98 | 2.98 | / | 3.46 |  |
| Z |  | 0.661 | / | 0.626 |  | 2.32 | / | 0.89 |  | 90.68 | / | 96.61 | 4.27 | / | 4.15 |  |
| a |  | 0.632 | / | 0.598 |  | 2.38 | / | 1.02 |  | 94.59 | / | 97.30 | 1.08 | / | 5.31 |  |
| b |  | 0.668 | / | 0.633 |  | 2.46 | / | 0.91 |  | 85.62 | / | 93.84 | 2.42 | / | 2.48 |  |
| c |  | 0.649 | / | 0.651 |  | 2.51 | / | 0.83 |  | 87.82 | / | 97.42 | 2.06 | / | 2.19 |  |
| d |  | 0.532 | / | 0.519 |  | 1.85 | / | 1.08 |  | 89.10 | / | 96.84 | 2.44 | / | 3.50 |  |
| e |  | 0.116 | / | 0.194 |  | 2.74 | / | 1.75 |  | 78.03 | / | 86.36 | -0.03 | / | 1.44 |  |
| f |  | 0.511 | / | 0.450 |  | 2.31 | / | 1.29 |  | 88.73 | / | 94.37 | 2.05 | / | 0.62 |  |
| g |  | 0.646 | / | 0.613 |  | 2.31 | / | 0.97 |  | 91.34 | / | 96.85 | 3.59 | / | 3.06 |  |
| h |  | 0.454 | / | 0.452 |  | 2.43 | / | 1.06 |  | 85.42 | / | 94.79 | 1.76 | / | 1.83 |  |
| i |  | 0.686 | / | 0.667 |  | 2.81 | / | 1.20 |  | 85.11 | / | 91.49 | 4.61 | / | 3.76 |  |
| j |  | 0.623 | / | 0.621 |  | 2.08 | / | 0.97 |  | 95.18 | / | 97.59 | 3.82 | / | 3.42 |  |
| k |  | 0.322 | / | 0.453 |  | 3.43 | / | 0.74 |  | 78.05 | / | 98.78 | 3.36 | / | 2.83 |  |
| o |  | 0.621 | / | 0.598 |  | 2.73 | / | 0.80 |  | 86.96 | / | 95.65 | 2.83 | / | 2.36 |  |
| p |  | 0.567 | / | 0.543 |  | 1.98 | / | 1.21 |  | 92.41 | / | 92.41 | 2.46 | / | 1.55 |  |
| q |  | 0.471 | / | 0.477 |  | 1.82 | / | 0.66 |  | 95.24 | / | 98.41 | 2.40 | / | 2.40 |  |
| r |  | 0.640 | / | 0.605 |  | 3.00 | / | 1.03 |  | 89.29 | / | 94.29 | 3.73 | / | 4.60 |  |
| s |  | 0.688 | / | 0.670 |  | 2.68 | / | 1.26 |  | 89.07 | / | 93.44 | 2.20 | / | 1.49 |  |
